# Local Environmental Conditions Shape Generalist But Not Specialist Components of Microbial Metacommunities in the Baltic Sea

**DOI:** 10.3389/fmicb.2016.02078

**Published:** 2016-12-23

**Authors:** Markus V. Lindh, Johanna Sjöstedt, Michele Casini, Agneta Andersson, Catherine Legrand, Jarone Pinhassi

**Affiliations:** ^1^Centre for Ecology and Evolution in Microbial Model Systems, Linnaeus UniversityKalmar, Sweden; ^2^Department of Aquatic Resources, Institute of Marine Research, Swedish University of Agricultural Sciences (SLU)Lysekil, Sweden; ^3^Department of Ecology and Environmental Science, Umeå UniversityUmeå, Sweden

**Keywords:** metacommunity, assembly mechanism, net relatedness index, niche breadth, generalist, specialist, habitat filtering

## Abstract

Marine microbes exhibit biogeographical patterns linked with fluxes of matter and energy. Yet, knowledge of the mechanisms shaping bacterioplankton community assembly across temporal scales remains poor. We examined bacterioplankton 16S rRNA gene fragments obtained from Baltic Sea transects to determine phylogenetic relatedness and assembly processes coupled with niche breadth. Communities were phylogenetically more related over time than expected by chance, albeit with considerable temporal variation. Hence, habitat filtering, i.e., local environmental conditions, rather than competition structured bacterioplankton communities in summer but not in spring or autumn. Species sorting (SS) was the dominant assembly process, but temporal and taxonomical variation in mechanisms was observed. For May communities, Cyanobacteria, Actinobacteria, Alpha- and Betaproteobacteria exhibited SS while Bacteroidetes and Verrucomicrobia were assembled by SS and mass effect. Concomitantly, Gammaproteobacteria were assembled by the neutral model and patch dynamics. Temporal variation in habitat filtering and dispersal highlights the impact of seasonally driven reorganization of microbial communities. Typically abundant Baltic Sea populations such as the NS3a marine group (Bacteroidetes) and the SAR86 and SAR11 clade had the highest niche breadth. The verrucomicrobial *Spartobacteria* population also exhibited high niche breadth. Surprisingly, variation in bacterioplankton community composition was regulated by environmental factors for generalist taxa but not specialists. Our results suggest that generalists such as NS3a, SAR86, and SAR11 are reorganized to a greater extent by changes in the environment compared to specialists and contribute more strongly to determining overall biogeographical patterns of marine bacterial communities.

## Introduction

Understanding the mechanisms that regulate microbial distribution patterns is a central objective in microbial ecology since microorganisms determine dynamics in processing of elements essential to life (Falkowski et al., 2008; Gomez-Consarnau et al., 2012; Logue et al., 2015). Yet, despite the recognized importance of microbial biogeography (Pommier et al., 2007; Barberan and Casamayor, 2010; Ghiglione et al., 2012), the assembly processes involved in structuring bacterioplankton communities are poorly understood (Martiny et al., 2006; Pommier et al., 2007; Barberan and Casamayor, 2010; Ghiglione et al., 2012; Lindström and Langenheder, 2012). However, current advancements in high-throughput sequencing now offer an opportunity for microbial ecologists to introduce and test mechanistic concepts in microbial biogeography (Poisot et al., 2013).

Bacterioplankton communities may be structured both by local and regional factors. The net relatedness index (NRI) measures species relatedness within a local community and estimates the importance of environmental conditions versus competition (Webb, 2000). A positive NRI value can be interpreted as habitat filtering, where members of the community are more closely related than expected by chance, indicating that community composition is structured by local environmental conditions (Webb et al., 2002). In contrast, a negative value implicates competitive exclusion of closely related species, leading to a local community with more distantly related species. Still, competitive exclusion assumes that communities are at steady-state (Rescigno and Richardson, 1965; Armstrong and McGehee, 1980), and steady-state in natural assemblages may rarely be achieved depending on, e.g., varying time scales of mixing/disturbances compared to growth rates. There is limited data on phylogenetic relatedness among microbial assemblages in marine systems and most have found positive NRI values, suggesting that habitat filtering (environmental factors) is important for bacterioplankton community structure (Barberan and Casamayor, 2010; Pontarp et al., 2012). Nevertheless, the extent of temporal variation in phylogenetic relatedness among bacterioplankton assemblages remains little studied.

Metacommunity theory predicts the interdependence of local environmental interactions and dispersal-driven processes (Mouquet and Loreau, 2003; Leibold et al., 2004; Holyoak et al., 2005; Beisner et al., 2006). There are currently four conceptual paradigms of metacommunity theory. Species sorting (SS) indicates that local environmental conditions regulate community structure whereas mass effect (ME) and patch dynamic (PD) indicate dispersal-driven assortment of communities. The neutral model (NM), in turn, emphasizes the importance of stochastic assembly processes (Logue et al., 2011). To our knowledge, three studies examining assembly mechanisms of bacterioplankton communities have been performed in marine environments; in the southern East China Sea, among *Vibrio cholerae* strains collected around the central California coast, and for 16 mainly coastal sites distributed globally (Keymer et al., 2009; Barberan and Casamayor, 2010; Yeh et al., 2015). In contrast, limnic environments are better understood (see, e.g., Beisner et al., 2006; Van der Gucht et al., 2007; Lindström et al., 2010; Logue and Lindström, 2010; Langenheder et al., 2012; Lindström and Langenheder, 2012; Adams et al., 2014). Collectively, examination of assembly mechanisms of bacterioplankton communities in aquatic environments indicates that SS (i.e., local environmental conditions) is the main driver of bacterial community structure. Nevertheless, although local environmental conditions are the dominant factor in shaping bacterioplankton communities, both Langenheder et al. (2012) and Yeh et al. (2015) observed temporal changes in assembly processes. Thus, information is lacking on the factors affecting bacterioplankton assembly mechanisms, including the magnitude and prevalence of temporal changes, or the influence of different taxa-intrinsic characteristics such as dispersal capacity, or a generalist versus specialist nature (Lindström and Langenheder, 2012).

Our aim was to examine assembly processes for structuring bacterioplankton community composition and biogeography using samples collected from monthly transects during April to October 2011 along a 100-km transect off the east coast of Sweden in the western Gotland Sea of the Baltic Sea Proper (Diaz-Gil et al., 2014; Legrand et al., 2015; Bertos-Fortis et al., 2016). Using 16S rRNA gene fragments we investigated (i) phylogenetic relatedness over time, (ii) differences in community assembly mechanisms over time, and between taxa, and (iii) how niche breadth influenced community assembly. Firstly, we hypothesized that substantial temporal variation in phylogenetic relatedness and assembly processes would result from seasonal changes in environmental conditions. Secondly, we hypothesized that different major taxa would exhibit different assembly processes. A final hypothesis was that variation in local environmental conditions would significantly influence community composition for taxa with limited niche breadth (i.e., specialists) but not taxa with a wide niche breadth (i.e., generalists).

## Materials and Methods

### Sample Collection, Physicochemical Factors, and Processing of 16S rRNA Amplicons

We used samples collected from monthly transects during April to October 2011 along a 100-km transect off the east coast of Sweden in the western Gotland Sea of the Baltic Sea Proper (for a detailed description of the study area and environmental conditions, see Diaz-Gil et al., 2014; Legrand et al., 2015; Bertos-Fortis et al., 2016). In brief, unfiltered natural seawater was collected in acid washed Milli-Q rinsed polycarbonate bottles, at discrete depths (2, 4, 6, 8, and 10 m) that were pooled and filtered shipboard on to 47 mm 0.2 μm Supor filters (Pall corporation). In total 13 stations were sampled from April to October, a total 63 samples analyzed, representing seasonal variation of coastal and open ocean sites. Samples for measuring Chlorophyll *a* (Chl *a*) concentration were collected according to Jespersen and Christoffersen (1987), and dissolved inorganic nutrients (NH_4_^+^, NO_3_^-^, PO_4_^3-^, and SiO_2_) were analyzed following the method of Valderrama (1995; for details on sampling abiotic factors, see Legrand et al., 2015; Bertos-Fortis et al., 2016). Sampling collection, DNA extraction, PCR amplification, and amplicon processing is detailed in Bertos-Fortis et al. (2016). Collection and extraction of DNA was performed according to Riemann et al. (2000). Bacterial 16S rRNA was amplified with bacterial primers 341F and 805R targeting the V3–V4 hypervariable region and containing adaptor and barcode following the protocol of Herlemann et al. (2011). The resulting purified barcoded amplicons were normalized in equimolar amounts and sequenced on a Roche GS-FLX 454 automated pyrosequencer (Roche Applied Science, Branford, CT, USA) at SciLifeLab, Stockholm, Sweden. Raw sequence data generated from 454 pyrosequencing were processed following Quince et al. (2011) and taxonomically identified using the SINA/SILVA database (Quast et al., 2013). Sequences were clustered together into operational taxonomic units (OTU) at the 97% 16S rRNA gene identity level using Usearch (Edgar, 2010). For subsequent analyses all samples were rarefied to 2500 sequences per sample. Amplicon sequences from the 16S rRNA gene fragments obtained from Bertos-Fortis et al. (2016) were deposited in the National Center for Biotechnology Information (NCBI) Sequence Read Archive under accession number SRP023607.

### Statistical Tests

A maximum likelihood-based phylogenetic tree for analyzing NRI was calculated using MEGA 5.0 (Tamura et al., 2011) using nearest neighbor interchange. Calculations for NRI are detailed in Webb (2000) and Horner-Devine and Bohannan (2006).

Correlations between bacterioplankton community composition (Bray–Curtis distances) and environmental factors versus spatial factors (Euclidean distances of salinity, temperature, Chl *a*, ammonium [NH_4_^+^], nitrate [NO_3_^-^], phosphate [PO_4_^-^], and silicate [SiO_4_] compared to latitude and longitude) were calculated using standard Mantel’s tests for environmental [E] and spatial [S] factors and partial Mantel’s tests for the fraction of bacterioplankton community composition that can be explained by the environmental factors independent of any spatial structure [E| S] and the fraction that can be explained by spatial allocation independently of any environmental variables [S| E] (**Table 1**). Assignment to the most appropriate metacommunity type was performed following Cottenie (2005).

**Table 1:** Summary of Mantel’s tests performed in the present study.

Month (*n* = 7)	Group (*n* = 7)	Number of Mantel’s tests
All	All	1
Separate months	All	7
All	Separate groups	7
Separate months	Separate groups	49

Niche breadth was calculated using Levin’s niche breadth index following Pandit et al. (2009) (*B* = 1/Σ*^N^_i_* = 1p^2^*_ij_*) where p*_ij_* is the proportion of OTU *j* in the sample *i* and *N* is the number of samples. OTUs with high *B* values are classified as habitat generalists and evenly distributed along a wide range of habitats. In contrast, OTUs with low *B* values are considered habitat specialists and unevenly distributed among sampling sites. Mantel’s tests for [E] and [S] and partial Mantel’s tests for [E| S] and [S| E] was performed for these different niche breadth groups as above.

All statistical tests and graphical outputs were performed in R 3.2.2 (R Development Core Team, 2014), using the packages Vegan (Oksanen et al., 2010), picante (Kembel et al., 2010), and ggplot2 (Wickham, 2009).

## Results and Discussion

### Importance of Environmental Conditions and Competition within a Local Community

To determine the importance of habitat filtering compared to competition for shaping local community composition we analyzed the phylogenetic relatedness among OTUs (conservatively defined at 97% 16S rRNA gene identity). Using the NRI index, 49 of 63 values were positive while 14 were negative (**Table 2**). From the 49 positive NRI values, 14 were significantly positive and most of these values (11 of 14) were found in July and August (**Table 2**). Positive NRI values are in agreement with previous studies indicating that local environmental conditions are important for community structure (Andersson et al., 2010; Barberan and Casamayor, 2010; Pontarp et al., 2012). Nevertheless, to our knowledge ours is the first study that has investigated temporal variations in bacterial phylogenetic relatedness.

**Table 2:** Phylogenetic relatedness over time.

	Month	+ (*n*)	- (*n*)	+ (%)	- (%)	Sig + (*n*)	Sig - (*n*)	Sig + (%)	Sig - (%)
	April	3	4	42.9	57.1	0	0	0	0
	May	3	8	27.3	72.7	0	0	0	0
	June	4	1	80	20	1	0	20	0
**NRI**	July	10	1	90.9	9.1	7	0	63.6	0
	August	12	0	100	0	4	0	33.3	0
	September	12	0	100	0	1	0	8.3	0
	October	5	0	100	0	1	0	20	0

*Maximum likelihood-based phylogenetic tree for analyzing NRI was calculated from MEGA 5.0 (Tamura et al., 2011) using nearest neighbor interchange. Number of and percentage of positive and negative NRI values is indicated with ± (*n*) and ± (%), respectively. Number of and percentage of significantly positive or negative NRI values is indicated with Sig ± and Sig ± (%), respectively*.

The composition of local bacterioplankton communities is generally dictated by biogeographical distribution and dispersal capability of populations, local adaptive radiation, intra- and inter-specific interactions, and local environmental effects resulting from changes in physicochemical conditions such as temperature, salinity, and nutrient availability (Webb, 2000). Although these processes can act synergistically, their relative importance varies over spatial, temporal, and phylogenetic scales (Martiny et al., 2006; Hanson et al., 2012; Lindström and Langenheder, 2012). Pontarp et al. (2012) proposed that despite the recognized temporal variation in bacterioplankton community composition (see, e.g., Andersson et al., 2010), the dominating assembly processes are similar. Moreover, the study performed by Andersson et al. (2010) showed an inverse correlation between genetic distance and similarity in OTU abundance profiles. The authors suggested that closely related taxa have coherent temporal dynamics and share similar ecological niches. Collectively, these studies point toward phylogenetic conservatism of functional traits among microorganisms. However, as Martiny et al. (2013) pointed out, phylum and class level conservation of traits generally appears to be limited. We show a considerable temporal variation in phylogenetic relatedness (**Table 2**), implying that multiple mechanisms can shape microbial communities across time. These results suggest that habitat filtering only structures marine bacterial communities under certain conditions. Phylogenetic conservatism likely influences the dynamics of the bacterial communities but the effects are masked at the community level since traits are more conserved at higher taxonomical ranks such as genus and/or species. Still, care should be taken when making conclusions on samples obtained at a single site or over a larger geographical area on one occasion, as the dynamics of bacterioplankton community assortment are instead largely dictated by the prevailing mechanism at any given time and changes seasonally.

### Importance of Local Environmental Conditions and Dispersal for Differences in Community Structure among Sites

**FIGURE 1:**
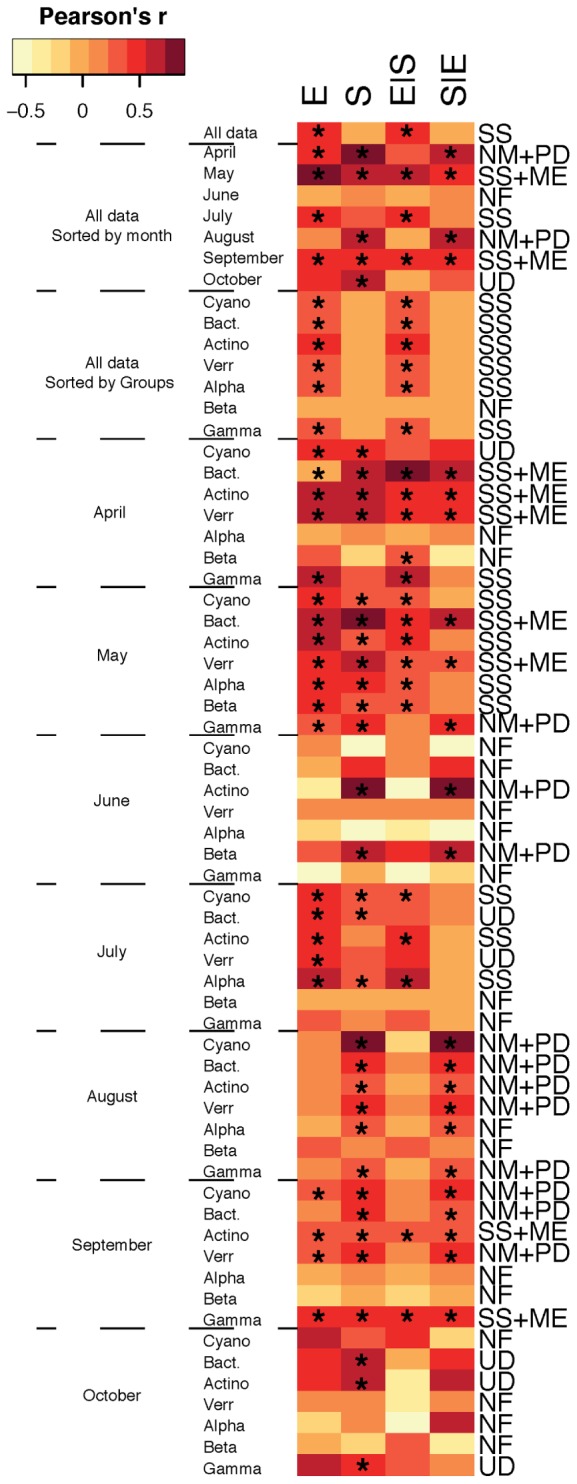
**Metacommunity types found in our study**. Variation in bacterial community composition was split into the following components: [E] environmental, [S] spatial, [E| S] the fraction of bacterial community composition that can be explained by the environmental factors independent of any spatial structure, and [S| E] the fraction of bacterioplankton community composition that can be explained by spatial allocation independently of environmental variables. Color in heatmap indicates Pearson’s *r* correlation and asterisk (^∗^) indicates significant values. Metacommunity types are abbreviated; SS, species sorting; ME, mass effect; NM, neutral model; PD, patch dynamic; UD, undetermined; NF, not found.

When assembly mechanisms were examined for the total bacterial community for all months together, SS was the main assembly mechanism (**Figure 1**) and analysis of the different bacterial taxa for all months together showed that SS remained the predominant assembly process. Indeed, SS has previously been emphasized as the principal assembly mechanism structuring bacterioplankton communities (Beisner et al., 2006; Van der Gucht et al., 2007; Barberan and Casamayor, 2010; Lindström et al., 2010; Logue and Lindström, 2010; Lindström and Langenheder, 2012) and Baltic Sea bacterioplankton communities have been shown to be largely structured by changes in salinity (Herlemann et al., 2011; Dupont et al., 2014). In fact, a recent metagenomic study suggested a global brackish water microbiome exists (Hugerth et al., 2015). In addition, Baltic Sea bacterioplankton metacommunities have been shown to be shaped by seasonally anoxic conditions that promote redox-specialized bacterial populations (Laas et al., 2015). Altogether, studies highlight that local environmental conditions structure the regional distribution of bacterioplankton populations into distinct metacommunities. Yet, temporal changes in assembly mechanisms have also been demonstrated for bacterial communities in rock pools (Langenheder et al., 2012) and in the southern East China Sea (Yeh et al., 2015), and accordingly, assembly mechanisms estimated over time in the current dataset indicated differences between months (**Figure 1**), so that, for example, in April and August the total communities were structured according to both the NM and PDs while the May and September communities were structured by SS and ME. In conclusion, although the main assembly process was SS, there was at times a substantial effect of spatial factors in shaping community structure, indicating that dispersal-driven assembly processes were also important, and these results highlight seasonal variation in the assembly of microbial communities and indicate the need for studying temporal dynamics in greater detail to understand microbial metacommunity dynamics. Ultimately, we could potentially envision the use of seasonal shifts in local and regional distribution patterns of marine microbes to predict responses to anthropogenically induced climate change and shifts in carbon cycling in marine ecosystems.

Interestingly, we also found differences in metacommunity assembly processes for different major bacterial taxa within each month. For example, in May, Actinobacteria, Betaproteobacteria, Cyanobacteria, and Alphaproteobacteria were structured by SS, and Bacteroidetes and Verrucomicrobia exhibited SS and ME, whereas Gammaproteobacteria were structured by the NM and PDs (**Figure 1**). This emphasizes that there can be pronounced temporal differences in the assortment of bacterial communities and that different metacommunity paradigms vary in importance on seasonal scales and between taxa. Overall, these results are largely in agreement with assembly mechanisms observed for different major bacterial groups in globally distributed datasets in both lake and marine environments as well as rock pools located near the Baltic Sea (Barberan and Casamayor, 2010; Székely and Langenheder, 2014). It is, however, noteworthy that the NM and spatial effects were also found for Gammaproteobacteria in the study of Barberan and Casamayor (2010), but in contrast, Székely and Langenheder (2014) observed neither significant environmental nor spatial effects for this bacterial class. Nevertheless, it is notable that Gammaproteobacteria do not exhibit SS in these studies and only once within months in the present paper (April). The typically fast-growing gammaproteobacterial populations might be assembled by mechanisms other than SS since this class contains several opportunistic taxa that mainly enter and exit the rare and abundant compartments of bacterioplankton. Still, Gammaproteobacteria assemblages were assembled by SS for the overall study period (i.e., for all pooled months).

Collectively, distributions of bacterioplankton populations affiliated with Cyanobacteria and Alphaproteobacteria were mainly assembled by local environmental conditions whereas Bacteroidetes and Verrucomicrobia were structured by spatial and environmental effects. Assembly of Gammaproteobacteria was on the other hand largely determined by the NM in addition to spatial and environmental effects. These results suggest that the dominant Gammaproteobacteria class may be less sensitive to long-term changes in environmental conditions resulting from anthropogenically induced climate change. Hence, Gammaproteobacteria might have an increased importance in the microbial food web due to future ocean change. In contrast, Actinobacteria, Cyanobacteria, Alphaproteobacteria, Bacteroidetes, and Verrucomicrobia that were shaped by environmental and spatial factors are likely more sensitive to predicted long-term ocean changes in environmental conditions and dispersal limitation and might hence be replaced by opportunistic gammaproteobacterial taxa.

### Habitat Specialization versus Assembly Processes

**FIGURE 2:**
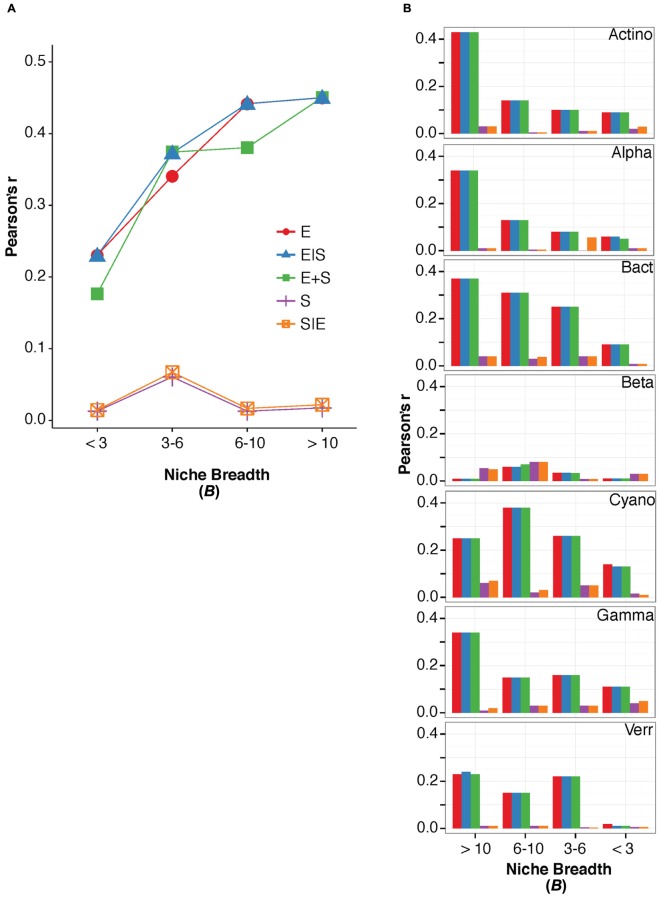
**The variation in bacterioplankton community composition that can be explained by the environmental and spatial factors within niche breadth calculated for all bacteria divided into groups of *B* > 10, *B* = 6–10, *B* = 3–6, and *B* < 3 (A)**, and within niche breadth calculated for specific bacterial groups **(B)**. Names of major bacterial groups are abbreviated; Actinobacteria (Actino), Alphaproteobacteria (Alpha), Bacteroidetes (Bact), Betaproteobacteria (Beta), Cyanobacteria (Cyano), Gammaproteobacteria (Gamma), Verrucomicrobia (Verr).

For terrestrial macroorganisms, community assembly is typically regulated by SS for habitat specialists, while habitat generalists are not significantly affected by changes in the environment (Leibold et al., 2004; Cottenie, 2005; Ellis et al., 2006). In comparison, zooplankton community assembly is regulated by SS for specialists (Pandit et al., 2009), while bacterial community composition can be explained by environmental factors for generalists (Székely and Langenheder, 2014). To examine how marine bacterioplankton generalists were assembled compared to specialists, we calculated the niche breadth (*B*), for the OTUs (Pandit et al., 2009). Most OTUs had a *B* < 3 (*n* = 1438) compared to the small number of OTUs with a *B* > 10 (*n* = 169; **Table 3**). Notably, over 40% of the total variation in community composition was explained by changes in environmental conditions for OTUs with *B* > 10 (**Figure 2**). In contrast, only around 20% of the total variation in community composition was explained by environmental factors for OTUs with *B* < 3 (**Figure 2A**). The OTUs with corresponding niche breadth (*B*) > 10 contributed to 40% of total sequences. In contrast, OTUs with *B* = 6–10 only contributed to 7% of total sequences, while OTUs with *B* between 3 and 6 and *B* < 3 contributed to 50% and 3% of the total sequence abundance, respectively. Notably, there was a significant positive correlation between community variance explained by environmental factors and niche breadth (linear regression, *p* = 0.04, *R*^2^ = 0.86; **Figure 2A**). This pattern was consistent among most bacterial groups except for Betaproteobacteria, Verrucomicrobia, and Cyanobacteria (**Figure 2B**). Thus, our results indicate that habitat generalists contribute substantially to determining spatiotemporal variation in marine bacterial community composition.

**Table 3:** Number of OTUs, average niche breadth (*B*) and occupancy for bacterioplankton communities within different ranges of niche breadth and within different bacterial taxa.

	Number of OTUs	Average *B*	Average occupancy (% of sites occupied)
All bacteria	2261	3.95 ± 5.33	8.56 ± 14.22
*B* > 10	169 (40%)	19.26 ± 8.8	49.45 ± 23.73
*B* = 6–10	188 (7%)	7.8 ± 1.14	17.70 ± 6.71
*B* = 3–6	467 (50%)	4.0 ± 0.85	7.63 ± 2.77
*B* < 3	1438 (3%)	1.62 ± 0.78	2.83 ± 1.28

Cyanobacteria	269 (13%)	4.28 ± 5.98	11.22 ± 19.47
Bacteroidetes	338 (20%)	4.99 ± 6.23	11.94 ± 17.55
Actinobacteria	663 (28%)	4.07 ± 5.99	8.35 ± 15.04
Verrucomicrobia	43 (3%)	5.67 ± 5.08	13.91 ± 18.04
Alphaproteobacteria	221 (14%)	4.40 ± 6.13	9.24 ± 15.64
Betaproteobacteria	65 (1%)	5.10 ± 5.53	10.72 ± 14.11
Gammaproteobacteria	125 (5%)	4.06 ± 5.48	9.15 ± 14.58

*Niche breadth was calculated for all taxa together but also for Actinobacteria, Bacteroidetes, Cyanobacteria, Firmicutes, Planctomycetes, Alphaproteobacteria, Betaproteobacteria, and Gammaproteobacteria separately, using Levins’ niche width (B) index. For Number of OTUs, percentages of total sequences (relative abundance) are provided in parenthesis*.

For individual OTUs we observed that typically numerically abundant lineages displayed high niche breadth, such as SAR11 OTU_41 and SAR86 OTU_7, with *B* = 40.58 and *B* = 45.02, respectively (**Table 4**). Yet, interestingly, other OTUs from the same clades were detected within *B* groups with lower niche breadth, e.g., SAR11 OTU_946 with *B* = 7.53, and SAR86 OTU_286 with *B* = 3.85. Typically, seasonally abundant populations in summer and autumn in the Baltic Sea Proper are exemplified by Verrucomicrobia and Actinobacteria, respectively (Lindh et al., 2015). The verrucomicrobial OTU_11 (*Spartobacteria*) were found within the group corresponding to *B* > 10 at 15.59. Two actinobacterial OTUs affiliated with the CL500-29 lineage (OTU_1248, OTU_2058) were found in the group with lowest niche breadth (*B* < 3). Collectively, our results indicate that taxa with a more restricted distribution range tended to be rare, i.e., with average relative abundances <0.1%. This indicates that most taxa with high niche breadth were common and abundant, while OTUs with lower niche breadth tended to be rare. Since we observed a significant correlation between niche breadth and the variance in community composition that was explained by environmental factors, we propose that habitat generalists such as SAR11 and SAR86 are likely to a greater extent affected by changes in environmental conditions. In agreement, previous studies have shown that habitat generalists respond to the major and strongest prevailing environmental conditions (Lennon and Jones, 2011; Székely and Langenheder, 2014).

**Table 4:** Niche breadth (*B*) of specific individual OTUs and their taxonomical affiliation.

	OTU	Phyla/class	Taxa	Niche breadth (*B*)	Average relative abundance (% total sequences)
	9	Bacteroidetes	NS3a marine group	45.39	2.88 ± 1.76
	7	Gammaproteobacteria	SAR86 clade	45.02	3.41 ± 2.23
	37	Actinobacteria	hgcI clade	43.19	1.3 ± 0.9
	47	Bacteroidetes	uncultured	41.69	0.71 ± 0.52
*B* top 10	41	Alphaproteobacteria	SAR11 clade	40.58	1.27 ± 0.95
	15	Alphaproteobacteria	SAR11 clade	39.58	1.64 ± 1.28
	42	Unclassified		38.63	1.14 ± 0.91
	8	Actinobacteria	hgcI clade	38.17	4.31 ± 3.52
	2	Alphaproteobacteria	SAR11 clade	37.03	6.46 ± 5.43
	32	Bacteroidetes	NS5 marine group	36.68	0.95 ± 0.82

	90	Bacteroidetes	*Fluviicola*	16.21	0.13 ± 0.21
	239	Actinobacteria	Microbacteriaceae	16.15	0.1 ± 0.18
	588	Alphaproteobacteria	SAR11 clade	15.75	0.03 ± 0.05
	11	Verrucomicrobia	LD29	15.59	1.37 ± 2.4
*B* > 10	77	Bacteroidetes	NS11-12 marine group	15.47	0.35 ± 0.61
	6	Cyanobacteria	FamilyI	15.41	0.87 ± 1.51
	211	Planctomycetes	*Planctomyces*	15.21	0.05 ± 0.08
	333	Alphaproteobacteria	Rhizobiales	15.04	0.03 ± 0.05
	248	Bacteroidetes	*Flavobacterium*	14.88	0.06 ± 0.12
	74	Alphaproteobacteria	Rhodobacteraceae	14.83	0.07 ± 0.12

	363	Verrucomicrobia	*Opitutus*	7.81	0.01 ± 0.03
	171	Bacteroidetes	*Fluviicola*	7.8	0.12 ± 0.33
	993	Actinobacteria	Micrococcales	7.78	0.02 ± 0.04
	1074	Actinobacteria	hgcI clade	7.74	0.01 ± 0.04
*B* = 6–10	119	Cyanobacteria	*Anabaena*	7.71	0.23 ± 0.62
	572	Bacteroidetes	*Flavobacterium*	7.68	0.02 ± 0.05
	454	Unclassified		7.67	0.03 ± 0.08
	427	Unclassified		7.57	0.02 ± 0.06
	946	Alphaproteobacteria	SAR11 clade	7.53	0.01 ± 0.03
	24	Cyanobacteria	*Anabaena*	7.52	1.14 ± 3.11
	212	Gammaproteobacteria	*Acinetobacter*	7.5	0.07 ± 0.02

	264	Bacteroidetes	*Algoriphagus*	3.92	0.01 ± 0.04
	268	Verrucomicrobia	FukuN18 freshwater group	3.91	0.05 ± 0.19
	987	Unclassified		3.9	0.01 ± 0.03
	1330	Actinobacteria	Sporichthyaceae	3.9	0.01 ± 0.03
	2005	Unclassified		3.86	0.01 ± 0.02
*B* = 3–6	495	Gammaproteobacteria	*Acinetobacter*	3.85	0.01 ± 0.03
	286	Gammaproteobacteria	SAR86 clade	3.85	0.01 ± 0.03
	613	Alphaproteobacteria	TK34	3.85	0.01 ± 0.03
	1715	Gammaproteobacteria	Idiomarinaceae	3.85	0.01 ± 0.03
	318	Verrucomicrobia	Marine group	3.79	0.01 ± 0.03

	999	Bacteroidetes	*Robiginitalea*	1.92	0.003 ± 0.02
	1248	Actinobacteria	CL500-29 marine group	1.92	0.003 ± 0.02
	1275	Planctomycetes	CL500-3	1.92	0.003 ± 0.02
	1331	Actinobacteria	uncultured	1.92	0.003 ± 0.02
*B* < 3	1403	Alphaproteobacteria	*Candidatus* Captivus	1.92	0.003 ± 0.02
	1499	Unclassified		1.92	0.003 ± 0.02
	1738	Cyanobacteria	FamilyI	1.92	0.003 ± 0.02
	1902	Bacteroidetes	*Owenweeksia*	1.92	0.003 ± 0.02
	2009	Verrucomicrobia	*Cerasicoccus*	1.92	0.003 ± 0.02
	2058	Actinobacteria	CL500-29 marine group	1.92	0.003 ± 0.02

	21578	Epsilonproteobacteria	*Arcobacter*	1	0.0007 ± 0.01
	21693	Actinobacteria	Microbacteriaceae	1	0.0007 ± 0.01
	21837	Gammaproteobacteria	*Psychrobacter*	1	0.0007 ± 0.01
	23354	Actinobacteria	Sporichthyaceae	1	0.0007 ± 0.01
	23821	Unclassified		1	0.0007 ± 0.01
*B* bottom 10	23972	Actinobacteria		1	0.0007 ± 0.01
	24642	Bacteroidetes	NS7 marine group	1	0.0014 ± 0.01
	25615	Betaproteobacteria	OM43 clade	1	0.0007 ± 0.01
	25676	Actinobacteria	hgcI clade	1	0.0007 ± 0.01
	25699	Unclassified		1	0.0007 ± 0.01

*The top 10 and bottom 10 OTUs are provided with the highest and lowest niche breadth, respectively. Ten OTUs were selected for each B group representing the median within that group. Average relative abundance (% of total sequences) with standard deviation is provided for each OTU*.

## Conclusion

Our results suggest that local environmental factors and SS are the major drivers of marine bacterioplankton community structure. Yet, assembly mechanisms vary over time. It remains unknown to what degree variability in these assembly processes depends on physical forcing, e.g., seawater is typically not stratified most of the year in the Baltic Sea, except for a strong stratification in summer. Further, assembly mechanisms vary substantially between different taxa within months and thus, multiple metacommunity assembly processes seem to concertedly structure microbial biogeography in the Baltic Sea Proper. We rejected our null-hypothesis that niche breadth, i.e., compositional shifts for habitat specialists, was not significantly explained by variation in environmental conditions. Collectively, these results highlight that generalists or common and widespread “core” taxa are sufficient to explain the overall observed patterns in beta-diversity as previously suggested based on research in rock pools by Székely and Langenheder (2014). Here we extend these findings to marine bacterioplankton suggesting that biogeographical patterns of marine bacteria are to a larger extent shaped by the “core” members of the community across environmental gradients compared to the rare “satellite” members. Members of the rare biosphere exhibit a substantial stochastic variation in their distribution across time and space, which complicates ambitions of predicting overall community structure and ultimately bacterial processing of carbon in a changing environment. Additionally, variations in rare OTUs may be more influenced than abundant OTUs by biases induced by varying sequencing depth between samples. Collectively, our results indicate that it would be preferable to focus on the common and widespread “core” community for understanding shifts in biodiversity patterns coupled with natural or anthropogenically induced changes in environmental conditions.

## Author Contributions

ML, CL, and JP conceived the study; ML, MC, CL, and JP designed research; ML and JS performed research; ML, JS, AA, and JP analyzed data; ML, JS, and JP wrote the paper. ML and JS contributed equally to this work. All authors discussed the results and commented on the manuscript.

## Conflict of Interest Statement

The authors declare that the research was conducted in the absence of any commercial or financial relationships that could be construed as a potential conflict of interest.
